# Supplementary material for: Clinical progression of clonal hematopoiesis is determined by a combination of mutation timing, fitness, and clonal structure
Source: bioRxiv. 2025 Mar 3:2025.02.28.640879. Preprint. [Version 2] doi: 10.1101/2025.02.28.640879 (PMC11908133; doi:10.1101/2025.02.28.640879)
Supplement: Supplement 1 [file NIHPP2025.02.28.640879v2-supplement-1.pdf]

(which was not certified by peer review) is the author/funder, who has granted bioRxiv a license to display the preprint in perpetuity. It is m[illegible]
available under aCC-BY 4.0 International license.

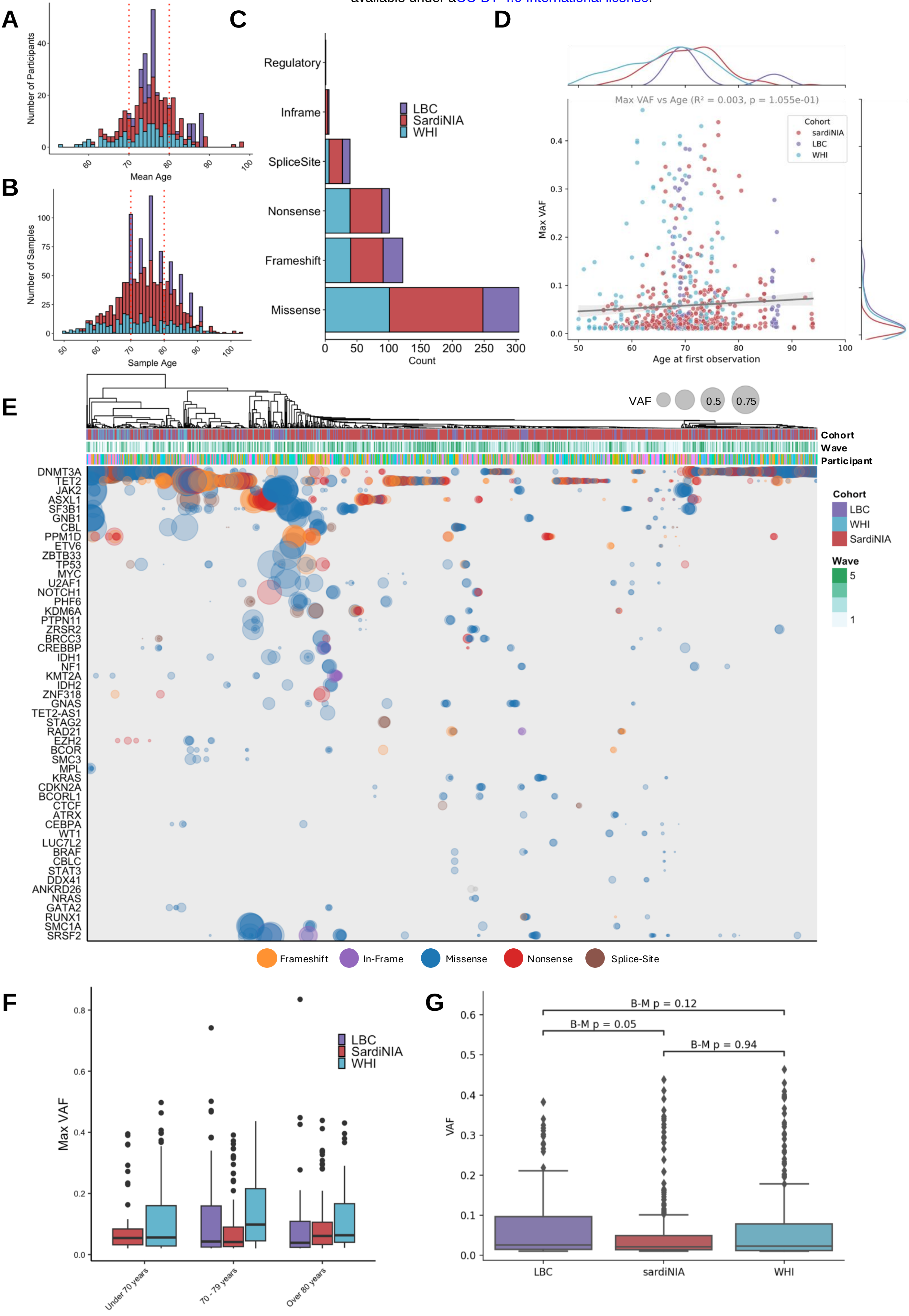

bioRxiv preprint doi: https://doi.org/10.1101/2025.02.28.640879; this version posted March 3, 2025. The copyright holder for this preprint (which was not certified by peer review) is the author/funder, who has granted bioRxiv a license to display the preprint in perpetuity. It is made available under aCC-BY 4.0 International license.

Figure S1: Complete cohort composition and overview.

A. Mean age of participants (across all measured longitudinal time points), colored by cohort.

B. Age of participants at sampling points, colored by cohort.

C. Counts of the functional consequences of variants, colored by cohort.

D. Age at the first observation of a given variant vs the maximum VAF measured across its trajectory, colored by cohort and flanked by cohort-normalized KDE curves on each axis. We observe a non-significant increase in maximum VAF (Max VAF) with age (R^2=0.03; p=1.055 e-01).

E. Variant allele fractions in all mutated genes, showing samples from all participants across all time points clustered by mutated gene. Here, each dot indicates the presence of the largest mutation within a gene for a given individual, which is scaled by VAF and colored by predicted functional consequence. The annotation bar indicates the participant, cohort, and wave number of samples.

F. VAF levels across all individuals at the first observed time point. Points are colored by cohort, with $\log_{10}$ scaled y-axis.

G. Distribution of maximum VAF (Max VAF) in each participant by cohort and age group. Data is split across age groupings that capture the under 70, 70-79 and 80 years and above. Boxes show the median and exclusive interquartile range with dots showing the outliers.

H. Distribution of maximum VAF (Max VAF) in each participant by cohort. Boxes show the median and exclusive interquartile range with dots showing the outliers. Statistical significance of VAF differences was assessed using the Brunner-Munzel test.

(which was not certified by peer review) is the author/funder, who has granted bioRxiv a license to display the preprint in perpetuity. It is m
available under aCC-BY 4.0 International license.

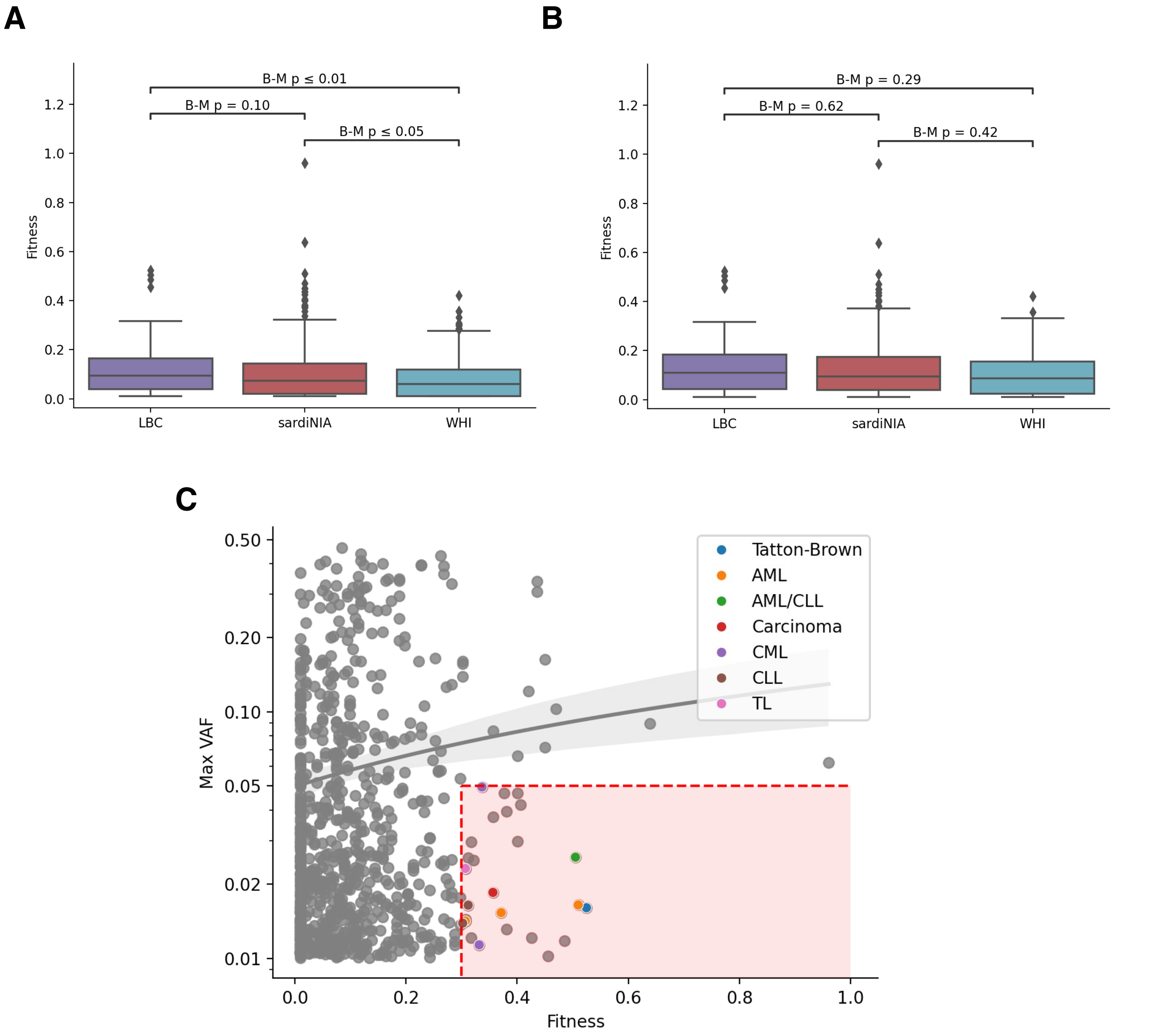

bioRxiv preprint doi: https://doi.org/10.1101/2025.02.28.640879; this version posted March 3, 2025. The copyright holder for this preprint (which was not certified by peer review) is the author/funder, who has granted bioRxiv a license to display the preprint in perpetuity. It is made available under aCC-BY 4.0 International license.

Figure S2: Cohort-specific analyses and mutation characteristics

A. Fitness for all mutations in each cohort. Boxes are colored by cohort and show exclusive interquartile range, with diamonds showing outliers. Statistical significance of fitness differences between cohorts was assessed using the Brunner-Munzel test.

B. Distribution of maximum fitness observed in each participant by cohort. Boxes are colored by cohort and show exclusive interquartile range, with diamonds showing outliers. Statistical significance of fitness differences between cohorts was assessed using the Brunner-Munzel test.

C. Maximum observed VAF of each mutational trajectory (shown on a log-scale) versus fitness. Colored points indicate variants reported for disease associations, with color showing which disease as shown in legend. Solid grey line and shaded region show linear regression fit showing a positive correlation between max observed VAF and fitness (R=0.1, p=0.002). Y-axis is log transformed to increase the visibility of low VAF variants. AML: Acute Myeloid Leukemia; CML: Chronic Myeloid Leukemia; CLL: Chronic Lymphoid Leukemia; TL: T-cell Lymphoma.

(which was not certified by peer review) is the author/funder, who has granted bioRxiv a license to display the preprint in perpetuity. It is m
available under aCC-BY 4.0 International license.

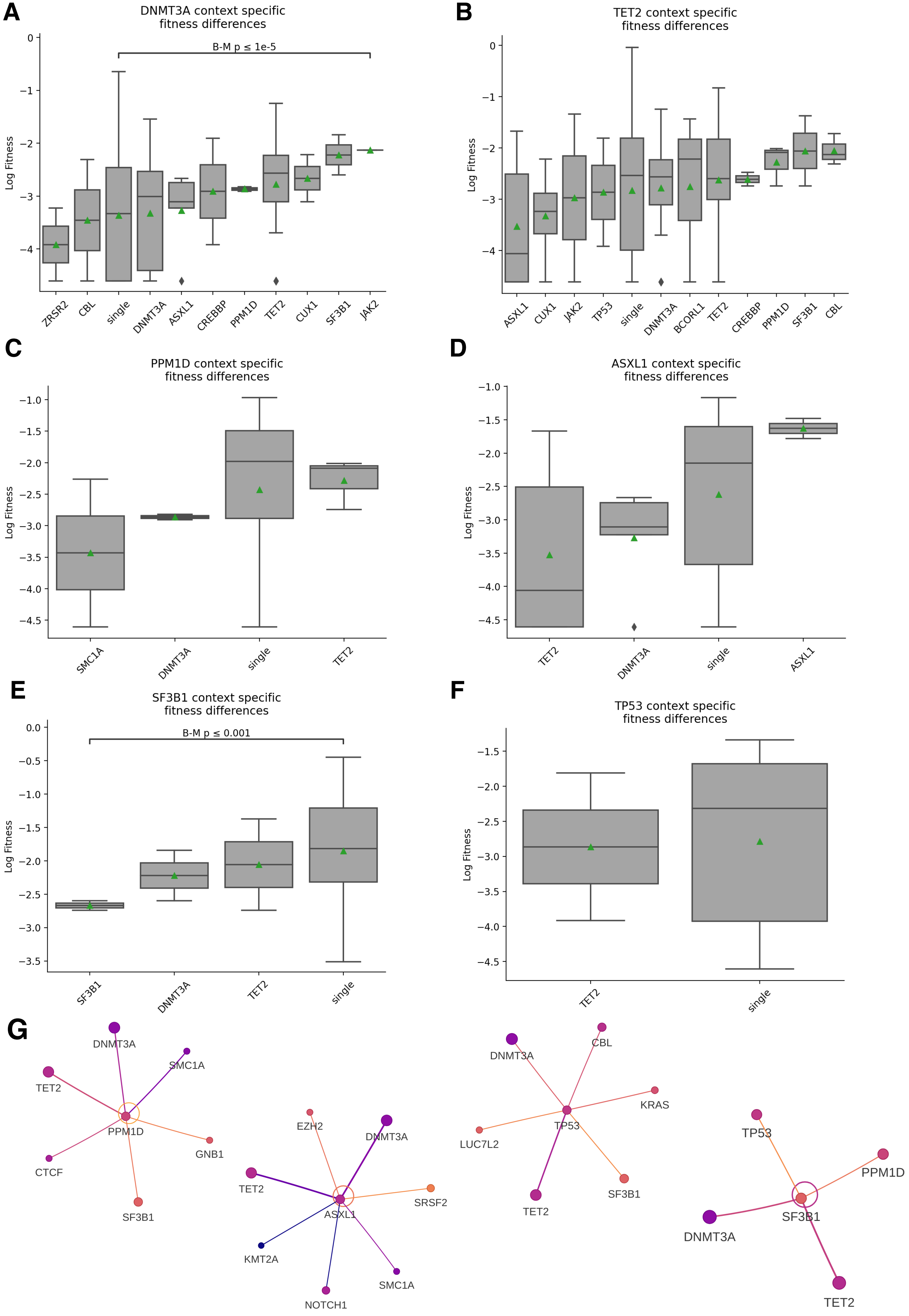

bioRxiv preprint doi: https://doi.org/10.1101/2025.02.28.640879; this version posted March 3, 2025. The copyright holder for this preprint (which was not certified by peer review) is the author/funder, who has granted bioRxiv a license to display the preprint in perpetuity. It is made available under aCC-BY 4.0 International license.

Figure S3: Differential fitness for different clonal compositions.

A-F. Differential fitness analysis of mutations in a reference gene (**A.** DNMT3A, **B.** TET2, **C.** PPM1D, **D.** ASXL1, **E.** SF3B1, **F.** TP53) across different combinations of co-mutated genes. For each reference gene we show boxplots comparing the log fitness of singly occurring mutations in a clone and co-occurring with mutations in other genes (only shown if more than 1 instance is observed). Boxes show median and exclusive interquartile range and diamonds show outlier values. Statistical significance of fitness differences between isolated and co-occurring mutations was assessed for log fitness using the Brunner-Munzel test with Benjamini-Yekutieli correction for multiple comparisons.

G. Network visualization showing mutation co-occurrence patterns for a single central gene. This representation is a direct isolation of the network associated to a single node as displayed in Fig 3C. Nodes represent individual mutations, with node size proportional to log counts of mutation instances and node color indicating average mutation log fitness (scaled between minimum and maximum log fitness values). Edges connect co-occurring mutations in a clonal structure, with edge width proportional to co-occurrence log counts and edge color representing the average log fitness of connected mutations.

(which was not certified by peer review) is the author/funder, who has granted bioRxiv a license to display the preprint in perpetuity. It is m
available under aCC-BY 4.0 International license.

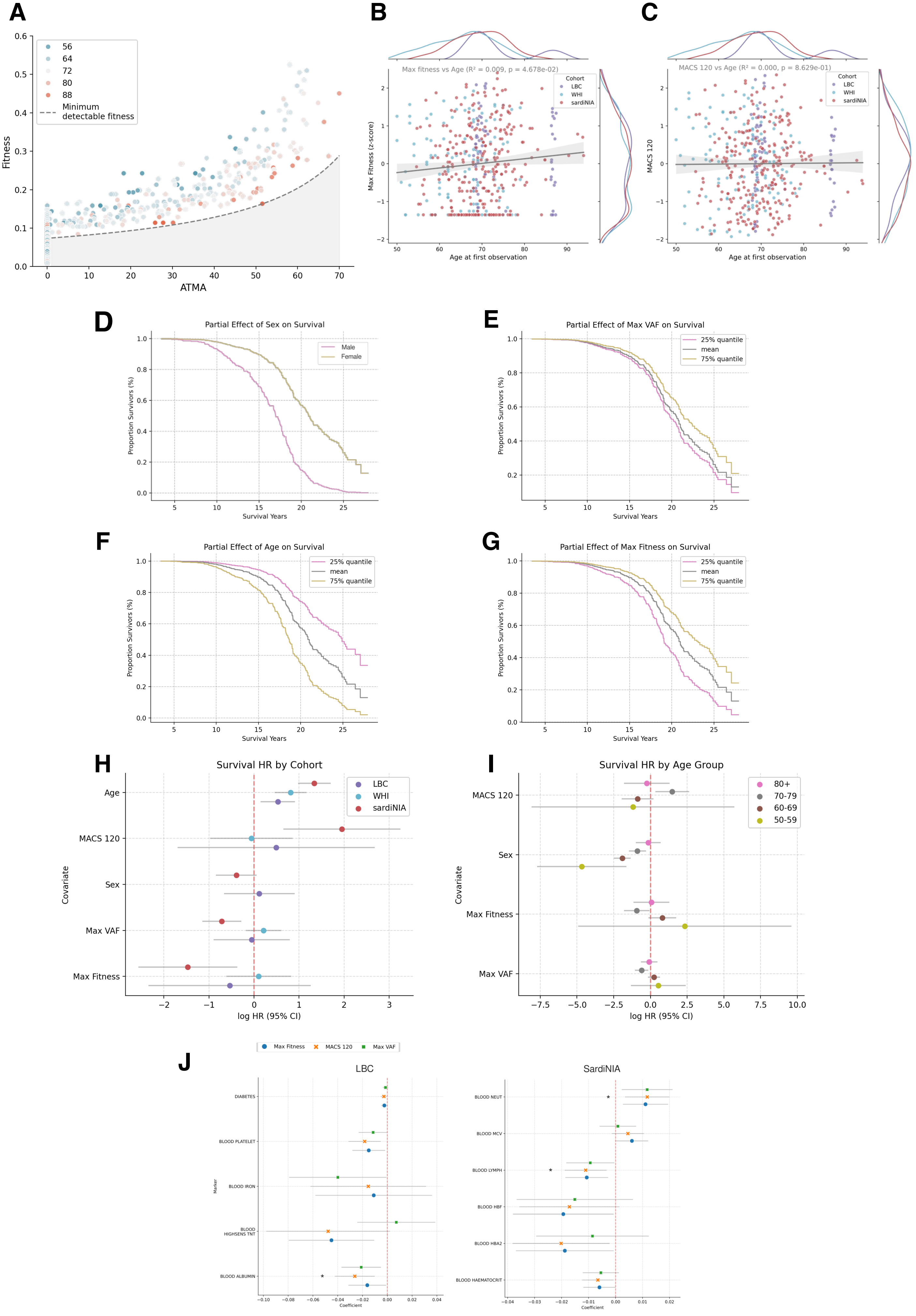

bioRxiv preprint doi: https://doi.org/10.1101/2025.02.28.640879; this version posted March 3, 2025. The copyright holder for this preprint (which was not certified by peer review) is the author/funder, who has granted bioRxiv a license to display the preprint in perpetuity. It is made available under aCC-BY 4.0 International license.

Figure S4: MACS120 and clinical outcomes.

A. Maximum fitness versus Age at Time of Mutation Acquisition (ATMA) with points colored by age of participant at first observation. The absence of low-fitness high-ATMA variants is due to detection limitations (dashed line, see Methods).

B. Correlation between maximum observed fitness in each individual and their age at first observation, with points colored by cohort. Grey line and shaded area show linear fit (R^2 =0.009, p=0.04). Marginal distributions shown as cohort-normalized Kernel Density Estimation (KDE) curves on each axis.

C. Correlation between MACS120 in each individual and their age at first observation, with points colored by cohort. Grey line and shaded area show linear fit (R^2 =0, p=0.86). Marginal distributions shown as cohort-normalized Kernel Density Estimation (KDE) curves on each axis.

D-G Partial effects in survival probability over time of model parameters included in the survival analysis. Parameters analyzed are: **D.** Sex, **E.** Maximum observed VAF in participant (z-score normalized) **F.** Age of participant at first observation (z-score normalized) **G.** Maximum observed fitness in participant (z-score normalized))**.** For each parameter, we show how variations in their values (25% quantile, baseline and 75% quantile) influence survival trajectories while controlling for other variables.

J. Cohort-specific analysis of the association between Maximum Fitness (blue dot), Maximum VAF (green square) and MACS120 (orange cross) and longitudinal changes in blood markers at the individual level. For each blood marker only metrics that showed a significant correlation are shown (see Methods). We display effect size and confidence intervals with a grey line. Acronyms: Lymphocytes (LYMPH) High-Sensitivity Cardiac Troponin T (HIGHSENS TNT), Mean Corpuscular Volume (MCV), Neutrophils (Neut), Thyroxine (T4).
